# What are the ethical implications of using prize-based contingency management in substance use? A scoping review

**DOI:** 10.1186/s12954-021-00529-w

**Published:** 2021-08-04

**Authors:** Marilou Gagnon, Alayna Payne, Adrian Guta

**Affiliations:** 1 Canadian Institute for Substance Use Research, University of Victoria, 2300 McKenzie Ave, Victoria, BC V8N 5M8 Canada; 2 School of Nursing, University of Victoria, 3800 Finnerty Road, Victoria, BC V8P 5C2 Canada; 3 School of Social Work, University of Windsor, 167 Ferry Street, Windsor, ON N9A 0C5 Canada

**Keywords:** Addiction, Contingency management, Ethics, Harm reduction, Incentives scoping review, Substance use

## Abstract

**Background:**

The area of substance use is notable for its early uptake of incentives and wealth of research on the topic. This is particularly true for prize-based contingency management (PB-CM), a particular type of incentive that uses a fishbowl prize-draw design. Given that PB-CM interventions are gaining momentum to address the dual public health crises of opiate and stimulant use in North America and beyond, it is imperative that we better understand and critically analyze their implications.

**Purpose:**

The purpose of this scoping review paper is to identify the characteristics of PB-CM interventions for people who use substances and explore ethical implications documented in the literature as well as emerging ethical implications that merit further consideration.

**Methods:**

The PRISMA-ScR checklist was used in conjunction with Arksey and O’Malley’s methodological framework to guide this scoping review. We completed a two-pronged analysis of 52 research articles retrieved through a comprehensive search across three key scholarly databases. After extracting descriptive data from each article, we used 9 key domains to identify characteristics of the interventions followed by an analysis of ethical implications.

**Results:**

We analyzed the characteristics of PB-CM interventions which were predominantly quantitative studies aimed at studying the efficacy of PB-CM interventions. All of the interventions used a prize-draw format with a classic magnitude of 50%. Most of the interventions combined both negative and positive direction to reward processes, behaviors, and/or outcomes. One ethical implication was identified in the literature: the risk of gambling relapse. We also found three emerging ethical implications by further analyzing participant characteristics, intervention designs, and potential impact on the patient–provider relationship. These implications include the potential deceptive nature of PB-CM, the emphasis placed on the individual behaviors to the detriment of social and structural determinants of health, and failures to address vulnerability and power dynamics.

**Conclusions:**

This scoping review offers important insights into the ethics on PB-CM and its implications for research ethics, clinical ethics, and public health ethics. Additionally, it raises important questions that can inform future research and dialogues to further tease out the ethical issues associated with PB-CM.

## Introduction

Over the past five decades, the use of incentives to modify health-related behaviors has generated a lot of interest in the fields of research, policy, and health care as well as in the private sector [1]. Incentives offer a solution to complex, persistent, and costly problems by increasing the appeal of adopting a new behavior and seeking to sustain that behavior over time [1]. Rooted in behavioral economics and behavioral psychology, incentives work at the psychological level but are primarily driven by economics—and the imperative to reduce both monetary costs (e.g., public spending, healthcare expenses, insurance claims, and so forth) and other costs associated with behaviors deemed “unhealthy” and “risky” (e.g., life expectancy, employee performance, health outcomes, disease burden, and so forth). Incentives have been used across clinical and public health areas to increase the appeal of behaviors such as clinic or group attendance, follow-ups, treatment adherence, immunization, screening, smoking cessation, weight loss or maintenance, healthy eating, exercise, substance use reduction or abstinence, and breastfeeding [2–15].

The area of substance use is notable for its early uptake of incentives and wealth of research on the topic. This is particularly true for contingency management (CM), a particular type of incentive that has gained popularity and has been used to reward behaviors such as group attendance, abstinence, and treatment adherence. CM draws on operant conditioning theory (also known as instrumental conditioning) and, more specifically, the general principle that behaviors such as substance use are encouraged or discouraged by their consequences [16]. To modify such behaviors, one has to modify the consequences using positive or negative reinforcements [17]. There are two types of CM, namely voucher-based CM and prize-based CM.^18^ Voucher-based CM provides a set monetary value in the form of vouchers that can be exchanged for items or gift cards of the same value [18]. However, this type of CM intervention is typically deemed too expensive [18]. Prize-based CM (henceforth PB-CM), also called fishbowl CM, attempts to lower costs by incorporating probability and variability [18]. Simply put, not all slips in the fishbowl are winning slips and the value of the slips varies from low to high. A standard fishbowl contains 500 slips with about half of the slips being non-winning and featuring messages such as “good job” [18]. The remaining slips are divided into three categories: (1) the majority of the winning slips are valued at $1, (2) a small number of winning slips are valued at $20, (3) and finally there is invariably only one large value slip of $100 [18]. Like vouchers, winning slips can be exchanged for items or gift cards of the same value [19].

Prize-based CM has been studied among people using stimulants, opioids, cannabis, nicotine, benzodiazepines, alcohol, as well as people who use multiple substances [20]. Yet, despite showing some level of efficacy (albeit only short-term) and being praised by researchers as beneficial and cost-saving, it continues to be “the least implemented” of all empirically based interventions in substance use [20]. This is, in part, due to overall cost and logistics of implementing PB-CM, but it is also reflective of a lack in awareness and knowledge on the part of healthcare providers as well as their overall weariness in adopting this type of incentive due to ethical and ideological tensions [18, 20]. Building on recent study findings on the experience of service and healthcare providers using PB-CM in Canada [21, 22], which confirm that such tensions exist, and recognizing the need to engage in a more in-depth analysis of PB-CM, we conducted a scoping review and completed a two-pronged analysis. First, we analyzed the characteristics of PB-CM interventions published in the literature, and then, we identified the ethical implications discussed as well as emerging ethical implications that merit further consideration in the field of substance use. The goal of this paper is to present findings of this analysis and discuss their implications for substance use-related policy, research, and practice.

## Background

In the area of substance use, CM was first used in 1970s, but it gained traction following a series of three papers published by Stitzer and colleagues between 1979 and 1982 [23–25]. In 1979, Stitzer and her team conducted a study with eight clients enrolled in a methadone maintenance program who were also known to use illicit benzodiazepines [23]. Prior to starting the study, the clients were switched to prescribed benzodiazepines—20 mg of diazepam per day upon request, 10 mg ingested in front of a nurse, and 10 mg dispensed for later use. Clients would alternate between “contingent” and “noncontingent” weeks. During contingent weeks, they would receive take-home privileges (in the form of take-home doses of methadone or limited methadone dosage regulation) if they declined the benzodiazepines and did not use illicit benzodiazepines (confirmed via urine drug screening). From this study, Stitzer and her team concluded that clinic privileges could be used as intervention tools to promote behavior change and promote reduction in or cessation of benzodiazepine use—and coined the term “contingent reinforcement” to describe this approach [23]. Two things are clear from this study and the studies that followed. One, CM has been used among people who use substances for more than five decades now. Two, CM continues to be presented as a neutral intervention that seeks to improve outcomes with little attention given to ethics.

In the 1990s, Higgins and colleagues developed a voucher-based CM system while conducting research with people who use cocaine [26–29]. The goal of this system was to achieve cocaine abstinence by integrating contingency management procedures to counseling. To test their system, Higgins and his team recruited participants with an active cocaine dependence and assigned them to two groups: a standard care group and a CM group for a total of 24 weeks [26]. Between weeks 1 and 12, each participant assigned to the CM group who provided a negative urine sample earned points (each point valued at $0.25), which were recorded on a voucher. With each negative sample, points increased in value and so did the total value of the voucher. Consecutive negative urine samples was positively reinforced by bonus amounts ($10 per for three consecutive negative urine samples), whereas a positive urine sample was negatively reinforced by resetting the voucher to its original value of $2.50. At the end of the 12 weeks, a participant who had remained abstinent for 12 weeks could have a voucher totaling close to $1000. Items available for purchase included ski passes, fishing licenses, camera equipment, gift cards for restaurants, sports equipment, and continuing education material. Between weeks 13–24, Higgins and his team replaced the vouchers with $1 state lottery tickets.

This example not only serves to illustrate how voucher-based CM works, but also why it is so costly. This type of CM can lead to earnings exceeding $1000 with an average ranging between $500 and $600 [30, 31]. Notwithstanding the ethical issues that arise from the potential coercive power of high monetary value incentives, the cost of this type of CM intervention alone was enough to prevent widespread implementation in clinical and community settings [32, 33]. To reduce the costs of voucher-based CM, Petry and colleagues [31] developed PB-CM also known as fishbowl CM. In contrast to voucher-based CM, where participants receive guaranteed positive reinforcement upon meeting a target, PB-CM provides reinforcement in form of having a chance to draw from a prize bowl [31]. As mentioned above, a standard prize bowl contains 500 slips with about half (between 40 to 60%) of the slips being non-winning and featuring a reinforcement message such as “good job,” and the remaining slips being divided into small (about 40% at $1), medium (8–10% at $20), and one jumbo prize of up to 100$ [31]. Similar to voucher-based CM, winning slips can be exchanged for items available onsite [31].

The PB-CM literature focuses heavily on abstinence. PB-CM interventions have been shown to increase short-term abstinence among people who smoke tobacco [34, 35], people who use stimulants [34, 37–39], and people who use opiates [36, 38, 40]. However, its ability to increase long-term abstinence remains questionable [41]. The focus of PB-CM interventions on abstinence along with its short-term effectiveness has been previously identified as limitations by healthcare providers who work in the area of substance use [42, 43], especially those who do not practice in the USA [42, 44, 45]. The same is true for the lack of attention given to the ethical implications of using PB-CM especially with the most marginalized groups of people who use substances [44–47]. A recent study conducted with service and health providers in Canada echoes these issues and points to important tensions emerging from PB-CM when it is used in a harm reduction context and with people who experience chronic poverty, precarious housing, and concurrent complex health issues [21, 22]. Given that PB-CM interventions are gaining momentum to address the dual public health crises of opiate and stimulant use in North America, it is imperative that we better understand and critically analyze the ethical implications. We recognize that this scoping review may not capture all of the ethical implications related to PB-CM, but we believe it has the potential to generate important questions that clinicians, researchers, and policy-makers should be asking and discussing.

## Methods

The PRISMA-ScR checklist was used in conjunction with Arksey and O’Malley’s methodological framework to guide this scoping review [48]. We selected Arksey and O’Malley’s as our primary framework because it was compatible with the goals of this paper and has been used extensively to guide scoping reviews focused on ethics [49–54]. This framework consists of five stages.

### Stage 1: identify the research questions

Our scoping review was designed to answer the following question: What are the ethical implications of using prize-based contingency management with people who use substances? The population was limited to people who use substances, the concept of interest was PB-CM interventions studied to date, and the context included any substance use treatment and care facilities, programs, or services. To answer our question, we turned to the research literature to identify the main objectives and findings of PB-CM studies. We also analyzed the design of PB-CM interventions. If ethical implications were taken into account in the study design, findings, and discussion, we documented them. We also looked for emerging ethical implications that merit further consideration. Scoping reviews are particularly useful when trying to chart documented *and* emerging ethical issues associated with new or rapidly developing healthcare interventions, especially when these interventions generate debates in a field [for example, see [55–58]].

### Stage 2: identify relevant studies

We developed a search strategy in collaboration with a specialized librarian. Databases included were Cumulative Index for Nursing and Allied Health Literature (CINAHL), MEDLINE, and PsychInfo. Major Mesh heading, mesh headings, and key word searches were used in each database as well as the Boolean operators AND as well as OR to maximize results. In each of the three databases, the following search was conducted: (Major Mesh heading “contingency management” OR keyword “contingency management”), AND (Mesh Word “substance use” OR “substance abuse” OR “substance misuse”) OR (subject heading “substance use” OR “Substance abuse” OR “substance misuse”). Query logic within each database was used accordingly to combine searches and alter language to best coincide with each database. Expanders included applying equivalent subjects within each database. Limiters included English language and publication in a peer-reviewed journal. We did not apply a date limiter to our search because our primary objective was to scope the body of research on PB-CM and we already knew that it spanned two decades—as mentioned above, CM in the form of PB-CM interventions was only introduced at the turn of the century. It is worth noting, however, that we excluded the term “prize” from the search strategy to maximize results. The reason is that many studies that focus on this particular type of CM do not specifically include “prize” in the title, key words, or abstract.

### Stage 3: selecting studies

**Fig. 1:**
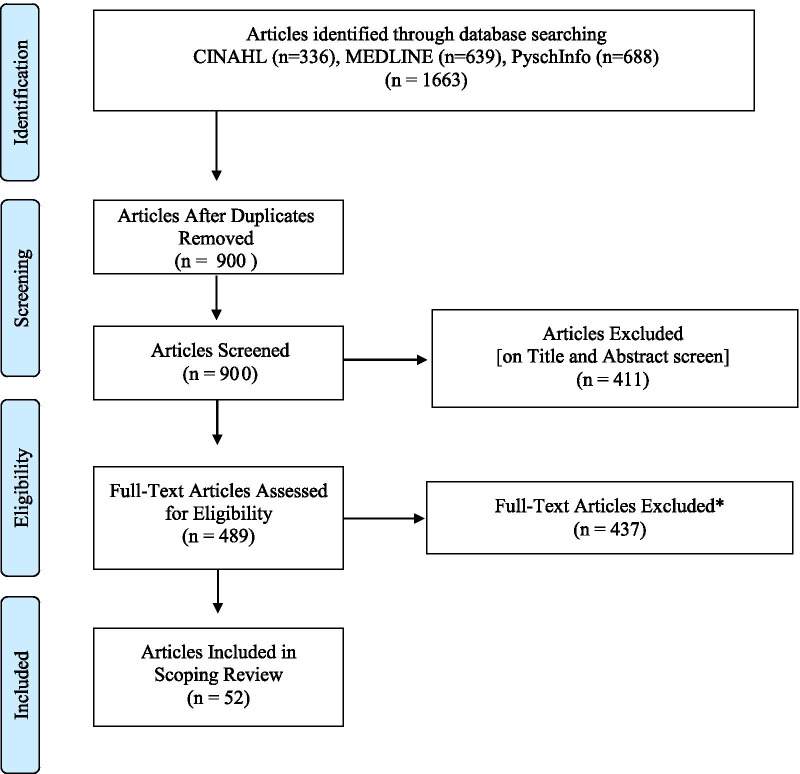
PRISMA flow diagram for literature search. *Reasons for exclusion: Wrong intervention (e.g., voucher-based CM) (*n* = 195) PB-CM is not the main focus of the article (*n* = 77); PB-CM is not the main intervention (*n* = 19); examined all types of CM together or did not explicitly identify PB-CM (*n* = 72); wrong design (e.g. editorials, book reviews) (*n* = 43); wrong outcome of interest (not looking at outcomes related to substance use) (*n* = 16); wrong indication (not implement to target substance use specific behavior) (*n* = 3); wrong patient population (*n* = 4); duplicates missed by citation management system (*n* = 8)

The search yielded 1663 articles, which we imported into Covidence. Once duplicates were removed, 900 articles remained. An initial title and abstract screen identified 411 studies that were not relevant to our scope. The titles and abstracts of the remaining 489 articles were re-screened by two authors (A.P. and M.G.) using the following inclusion and exclusion criteria. We included studies focusing on the implementation of PB-CM interventions designed to encourage people who use substances to take part in the treatment process (e.g., attending a group session), comply with prescribed behaviors (e.g., abstinence), and meet specific outcomes (e.g., negative urine drug screening). We did not apply limiters to the term “substances” as the type of substance was not relevant to our scope. We excluded articles that did not specify the type of CM intervention studied, articles that combined various types of CM interventions, articles where PB-CM was not the primary intervention of interest and those that examined PB-CM among populations other than people who use substances. We also excluded editorials, book reviews, and commentaries since the intention was to map out the existing body of research on PB-CM interventions. A total of 437 articles were excluded, leaving 52 articles to be included (see Fig. 1).

### Stage 4: charting data

We started by extracting descriptive data from each of the 52 articles sample and compiling it in an Excel spreadsheet using the following headings: authors, year of publication, geographic location, study objectives, target population, context, and study design. Then, we extracted data pertaining to the design of the PB-CM intervention using a framework of 9 key domains developed by Adams and colleagues (see Table 1) [59]. We chose this framework because it was developed to help researchers and clinicians describe incentive interventions. Finally, we documented ethical implications which were taken into account in the study design, findings, and discussion. We also identified emerging implications based on patient characteristics, intervention designs, and potential impact on the patient–provider relationship. To explore emerging implications, we drew from the documented experiences of service and healthcare providers with PB-CM [21, 22]. These experiences suggest that the specificities of PB-CM and the role providers play in the delivery of this intervention impacts the provider-patient relationship and give rise to ethical concerns pertaining to fairness, autonomy, power imbalance, and effectiveness [22]. We also applied a relational ethics lens to the analysis to reflect that ethics in PB-CM is shaped by provider characteristics, patient characteristics, contextual factors, and interpersonal dimensions [21].

**Table 1:** 9 key domains adapted from Adams et al. [59]

Domain	Description
Direction	Is the incentive a positive gain for meeting the target or avoidance of a negative loss for failing to meet such a target?
Form	What type of incentive is used? (e.g., cash, goods, services, vouchers, etc.)
Magnitude	What is the total value of the incentive offered?
Certainty	How certain are participants of receiving the incentive? Incentives are *certain* if they are automatically given upon meeting a target. In contrast, there is only a *certain chance* of receiving an incentive when delivered in the form of a standardized prize bowl and *uncertain chances* when delivered using a lottery-style incentive.
Target	What is the target of the incentive? Targets can include processes (e.g., attending a group session), behaviors (e.g., abstinence), and outcomes (e.g., negative urine drug screening).
Frequency	What proportion of target processes, behaviors, or outcomes are actually incentivized? Is the person receiving an incentive every time or some times?
Immediacy	What is the period between meeting a target and receiving the incentive?
Schedule	Is the schedule fixed or variable? Fixed schedule provide the same incentive every time the target is achieved while a variable schedule provides different (and often escalating) incentives. For example, a second negative urine drug screening warrants two draws, fourth negative urine sample warrants four draws and so forth.
Recipient	Is the recipient an individual, group, significant other, clinician, or parent?

### Stage 5: synthesizing and reporting the findings

Findings were organized in three main sections. The first section describes the sample. The second section presents the findings of the analysis using the 9 key domains framework [59]. The third section presents the findings of the ethical analysis and focuses on three main themes.

## Results

### Description of the sample

**Fig. 2:**
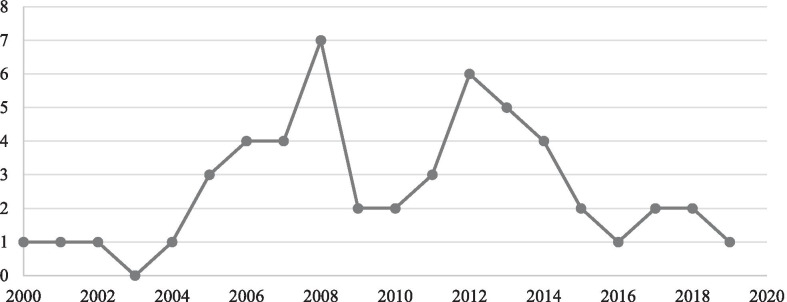
Number of prize-based contingency management articles published by year

More than a half (*n* = 28 or 53.8%) of our sample was authored or co-authored by the creator of PB-CM, psychologist Dr. Nancy M. Petry. Of our total sample, 24 articles had been published before 2010 and 28 after 2010, with two notable peaks in 2008 and 2012 (see Fig. 2). The vast majority of the articles originated in the USA (*n* = 49 or 94%).

Study objectives included (1) examining the efficacy of PB-CM interventions (*n* = 29) [30–32, 34–37, 39–41, 60–78], (2) assessing the cost-effectiveness of PB-CM (*n* = 4) [33, 79–81], (3) examining the feasibility of implementation PB-CM (*n* = 4) [82–85], (4) comparing PB-CM to voucher-based CM, or to standard care (*n* = 2) [86, 87], (5) examining the association between PB-CM and gambling (*n* = 2) [88, 89], (6) modifying and evaluating the intervention design (e.g., reinforcement schedule or magnitude) (*n* = 8) [40, 65, 90–95], (7) exploring strategies to increase the uptake or dissemination of PB-CM (*n* = 2) [96, 97], (8) or examining clinicians perceptions of PB-CM (*n* = 1) [98].The majority of our sample consisted of quantitative studies, mainly randomized controlled trials (*n* = 30 or 55.7%). The types of settings represented in the sample included inpatient and outpatient treatment and care facilities, programs, or services for people who use substances [30–37, 61–66, 68–72, 74–79, 82–87, 90–97]. They were invariably abstinence-based or recovery-oriented. This is exemplified in Table 2, which we will discuss in the next section, and reflected in the focus of the reward on abstinence-related behaviors and abstinence-related outcomes (e.g., a negative urine drug screening).

**Table 2:** Descriptive analysis based on 9 key domains [59] (*n* = 39)

Author	Date	Direction	Magnitude (%)	Certainty	Process	Behavior	Outcome	Frequency	Immediacy	Schedule	Recipient
1. Petry et al.	2000	+	75	Certain	X		X	All	Immediate	Variable	Individual
2. Petry et al.	2001	+	50	Certain	X	X		All	Immediate	Variable	Individual
3. Petry et al.	2002	+/−	50	Certain			X	All	Immediate	Variable (E-R)	Individual
4. Petry et al.	2004	+/−	50	Certain	X		X	All	Immediate	Variable (E-R)	Individual
5. Petry et al.	2005	+/−	50	Certain			X	All	Immediate	Variable (E-R)	Individual
6. Petry & Alessi et al.	2005	+/−	37.2	Certain	X		X	All	Immediate	Variable (E-R)	Individual
7. Petry & Martin et al.	2005	+/−	50	Certain		X	X	All	Immediate	Variable (E-R)	Individual
8. Peirce et al.	2006	+/−	50	Certain			X	All	Immediate	Variable (E-R)	Individual
9. Petry et al.	2006	+/−	45	Certain	X		X	All	Immediate	Variable (E-R)	Individual
10. Alessi et al.	2007	+/−	N/R^a^; 50^b^	Uncertain^a^; Certain^b^		X	X	Some^a^; All^b^	Immediate	Variable (E-R)	Individual
11. Petry et al.	2007	+/−	50	Certain			X	All	Immediate	Variable (E-R)	Individual
12. Ghitza et al.	2007	+/−	50	Certain			X	All	Immediate	Variable (E-R)	Individual
13. Alessi et al.	2008	+/−	100; 50*	Certain			X	Some	Immediate	Variable (E-R)	Individual
14. Kirby et al.	2008	+/−	50	Uncertain			X	Some	Immediate	Variable (E-R)	Group
15. Ledgerwood et al.	2008	+/−	100	Uncertain		X		Some	Immediate	Variable (E-R)	Individual
16. Preston et al.	2008	+/−	50	Certain			X	All	Immediate	Variable (E-R)	Individual
17. Lot et al.	2009	+/−	50	Certain		X	X	All	Delayed	Variable (E-R)	Individual
18. Olmstead et al.	2009	+/−	37.2	Certain		X	X	All	Immediate	Variable (E-R)	Individual
19. Walker et al.	2010	+/−	50^1^; 100^2^	Certain^1^; Uncertain^2^		X		All^1^; Some^2^	Immediate^1^ Delayed^2^	Variable (E-R)	Individual
20. Hser et al.	2011	+/−	50	Certain		X	X	All	Immediate	Variable (E-R)	Individual
21. Petry et al.	2011	+/−	100	Uncertain		X	X	Some	Immediate	Variable (E-R)	Individual
22. Branson et al.	2012	+	85	Certain		X		All	Immediate	Variable	Individual
23. Carroll et al.	2012	+	57.7	Certain	X		X	All	Immediate	Variable	Individual
24. Jiang et al.	2012	+	N/R	Uncertain			X	Some	Immediate	Variable	Individual
25. Killeen et al.	2012	+/−	60	Certain			X	All	Immediate	Variable (E-R)	Individual
26. Petry et al.	2012	+/−	50	Certain			X	All	Immediate	Variable (E-R)	Individual
27. Petry & Barry et al.	2012	+/−	50^1^ 100; 50*^2^	Certain	X		X	All	Immediate	Variable (E-R)	Individual
28. Chen et al.	2013	+/−	50	Certain	X	X	X	All	Delayed	Variable (E-R)	Individual
29. Hagedorn et al.	2013	+/−	50	Certain			X	All	Immediate	Variable (E-R)	Individual
30. McDonell et al.	2013	+/−	50	Certain			X	All	Immediate	Variable (E-R)	Individual
31. Petry et al.	2013	+/−	50	Certain			X	All	Immediate	Variable (E-R)	Individual
32. Roll et al.	2013	+/−	50	Certain			X	All	Immediate	Variable (E-R)	Individual
33. Alessi et al.	2014	+/−	100; 50*	Certain			X	Some	Immediate	Variable (E-R)	Individual
34. Ledgerwood et al.	2014	+/−	50	Certain			X	All	Immediate	Variable (E-R)	Individual
35. Petry et al.	2015	+/−	66; 33*^1^ 76; 50 *^2^	Certain			X	All	Immediate	Variable (E-R)	Individual
36. Cunningham et al.	2017	+/−	50	Certain	X		X	All	Immediate	Variable (E-R)	Individual
37. Kropp et al.	2017	+	N/R	Uncertain	X	X		Some	Delayed	Fixed	Individual
38. Petry et al.	2018	+/−	50	Certain	X	X		All	Immediate	Variable (E-R)	Individual
39. Rash et al.	2018	+/−	60	Certain			X	All	Immediate	Variable (E-R)	Individual

NR, Indicates not reported

^*^Indicates studies that used a “priming” technique where initial magnitude of drawing a winning prize card was higher followed by a lower magnitude, within the same group

^a,b^Indicates studies where one group was exposed to two distinct PB-CM interventions

^1,2^Indicates studies where two groups were used, where each group received a distinct PB-CM interventions

Four main target populations were identified. The most common target population was adults enrolled in or seeking treatment for substance use disorder, primarily through outpatient therapy or methadone maintenance therapy (*n* = 27). The remaining 3 target populations were (1) adults using one or more substances including nicotine, alcohol, stimulants, cannabis, and opiates (*n* = 17) [31, 33, 35, 39, 40, 60, 66, 67, 69, 73, 77, 88, 90–92, 95, 96]; (2) adults using substances with a concurrent mental health diagnosis (*n* = 4) [34, 36, 37, 81]; and (3) adolescents who either use substance use disorder or who are at risk of developing substance use disorder (*n* = 2) [32, 82] or adolescents attending treatment (*n* = 1) [62]. One study assessed PB-CM among people living with HIV attending substance use treatment groups at an outpatient facility [84].

### Characteristics of PB-CM interventions

Using the framework developed by Adams et al. [59], we identified the key characteristics of the PB-CM interventions, namely direction, form, magnitude, certainty, target, frequency, immediacy, schedule, and recipient (see Table 2). Only 39 of the 52 articles in our sample provide sufficient information for this analysis. We do not consider this to be a significant limitation given that PB-CM interventions share common features as shown in the following sections. However, it is an important gap in the literature, one that PB-CM researchers should address by providing more details on their intervention designs.

#### Direction

The majority of the studies used both a negative and positive direction in their intervention design (*n* = 33). This dual direction is identified by the +/− symbol in Table 2. A positive direction, in the form of a prize draw, was used to reward participants for meeting a target process, behavior, and/or outcome. A negative direction was used when the participants did not meet those targets resulting in either withholding prize draw(s) or resetting the amount of draws back to zero for interventions where cumulative draws were included in the design. The remaining studies used a solely positive direction (*n* = 6), meaning that participants were rewarded if they met the target and did not experience any losses if they did not beyond the missed opportunity to draw [20, 32, 61, 68, 69, 84].

#### Form

All of the studies used the prize draw method as the form of reinforcement. This is not a finding but rather a classic element of PB-CM intervention design in which having a chance to participate in the draw acts as the primary source of reinforcement. As such, we did not include form in Table 2 to avoid redundancy.

#### Magnitude

Typically, studies on PB-CM include a range of potential amounts and values of prizes that can be won by taking part in the intervention. In our sample, winning cards ranged from $1 to $100. However, this fixed range offers an incomplete picture of magnitude because it fails to account for the actual probability of drawing a winning card at every draw. Therefore, to extend our analysis of magnitude, we turned to the probability (in percentage) of drawing a winning card per draw. For example, if a prize bowl has 500 cards, and 250 of those cards have a monetary value, the probability of drawing a winning card is 50%. Considering that the majority of winning cards are small amounts, the probability of winning a high total sum (magnitude), such as $100, is actually quite low. The appeal of *potentially winning* that sum, however, is what drives this intervention. Only 37 of the 39 articles included sufficient information to document the probability of drawing a winning card per draw. Nearly three quarters of the sample (*n* = 28 or 72%) used a prize bowl that had a 50% probability of drawing a winning card per draw. To increase the appeal of potentially winning large sums, several studies (*n* = 10 or 26%) used a technique called “priming”—a term originally used by Higgins [26] and was later coined by Petry [21]. Priming refers to a process whereby the researchers boost the appeal at the start of the intervention by using two strategies. One priming strategy consists of using a higher magnitude prize bowl (80–100% probability) for the first several weeks followed by lower (standard) magnitude prize bowl (50% probability) for the remaining weeks [77, 78, 91, 93]. Another strategy consists of offering a guaranteed prize once a specific threshold is reached, such as completing two consecutive weeks of abstinence [30, 63, 79, 86, 87, 95].

#### Certainty

Of the 39 studies, nearly all of them (*n* = 24 or 61.5%) featured an intervention designed using a “certain chance” model. In other words, they were using a standardized prize bowl with the same probability of drawing a winning card at each singular draw. The remaining studies (*n* = 7 or 8 %) used an “uncertain chance” model [61, 65, 68, 71, 83, 85, 97]. Of note, two of these studies used a combination of uncertain and certain chance models [85, 97]. An example of an uncertain chance model is the use of PB-CM to incentivize attendance to a group therapy session [65]. Here, the structure of the intervention may involve two or more bowls, the first being a name bowl, where participants attending the session enter their name into, and the second being the prize bowl. At the end of the group session, the counsellor draws a name from the name bowl and that particular person then has a chance to draw from the prize bowl.

#### Target

For each study, we identified the target(s) and divided them into three categories: process, behavior, and outcome. Adams and colleagues [59] label the second category “intermediate,” but we refer to it as “behavior” in order to capture the nature of the target, which is individual behaviors. In our sample, examples of process targets included participation in activities with a clear emphasis on prevention and supports. Behavior targets included abstaining from using substances and treatment adherence such as attending treatment groups. Outcome targets included negative drug urine samples. Although several studies incentivized combinations of processes and/or behaviors with outcome targets (*n* = 15 or 38%) [31, 33, 62, 63, 65, 66, 69, 72, 74, 76, 79, 83, 85, 86, 91], there was an overwhelming focus on outcome targets as evidenced by 87% (*n* = 34) incentivizing outcome targets alone, or a combination of process and behavior targets with outcome targets. Nearly half of the sample (*n* = 19) focuses solely on incentivizing abstinence (outcome target). Contrastingly, only 12% (*n* = 5) of the studies focused on either process or behavior targets without incentivizing outcome targets [32, 61, 71, 95, 97].

#### Frequency

Nearly all articles incentivized the target every time it was reached (all) (*n* = 33). We found that PB-CM interventions that used certain chance models were designed to provide prize draws on all instances (*n* = 33), whereas interventions that used uncertain chance models were designed to reinforce some instances (*n* = 7) [61, 65, 68, 71, 83, 85, 97].

#### Immediacy

PB-CM interventions can be either immediate or delayed. In immediate interventions, participants access the prize draw immediately upon meeting a target. In delayed interventions, participants access the prize draw later such as at the end of the week or at the end of treatment. Only 10% (*n* = 4) of the interventions used a delayed reinforcement approach [61, 62, 72, 97].

#### Schedule

The schedule for all of the studies, with the exception of one, was a variable schedule reflecting the changing absolute value of reinforcements of the prize draw method. For example, the value of the prize varies based on the card(s) drawn (e.g., $1, $20, $100 or a non-monetary card with a positive encouraging message). In addition to using a variable schedule, 84.6% (*n* = 33) of the studies used an escalating and re-set technique (designated as E-R in table 2), which is consistent with the findings presented in the direction section. With this technique, participants can earn more draws as they continue the meet their target(s) (i.e., positive direction) and can have draws withheld or re-set back to one draw if they fail to do so (i.e., negative direction). Only 11% (*n* = 6) of the interventions omitted this negative component [31, 32, 61, 68, 69, 84].

#### Recipient

Recipients were individuals across all studies except for one, which was a group structure [83]. Within the group structure, the achievement of a target behavior by one randomly selected individual determines the fate of receiving an incentive for the entire group.

### Ethical implications

Across the sample (*n* = 52), we found that mentions of/or considerations for ethical implications were exceptionally sparse. We were able to identify one consistent ethical consideration across the sample: the potential risk of gambling relapse for patients in recovery from gambling . We also found three emerging ethical implications by further analyzing participant characteristics, intervention designs, and potential impact on the patient–provider relationship. These implications include the potential deceptive nature of PB-CM, the emphasis placed on the individual behaviors to the detriment of social and structural determinants of health, and failures to address vulnerability and power dynamics.

#### The risk of gambling relapse

**Fig. 3:**
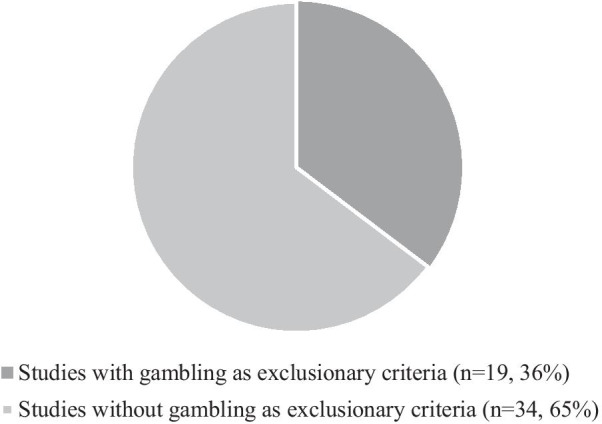
Proportion of studies with gambling exclusionary criteria

The exclusion of people in recovery from gambling was common across the literature. This is due to the potentially triggering effect of PB-CM on the conditional (dopamine) pathway involved in both substance use and gambling. In a standard PB-CM intervention, the appeal of the draw—and more importantly the probabilistic chance of winning the large value $100 slip—acts as a reward and makes the achievement of the target process, behavior, and outcome more appealing despite the actual monetary value of the incentive being quite low. Its efficacy relies primarily on the activation of the same pathway as gambling by leveraging the desire to draw the biggest slip. In our sample, more than a third of the articles (36%) mentioned the risk of increased gambling (see Figure 3) including 13 articles published after 2008. This finding is significant because Petry and colleagues [88] published a study in 2006 to challenges the idea that PB-CM can lead to a risk of increased gambling. In their study, which excluded people who were in recovery from gambling, Petry and her team [88] compared two randomized groups of people using substances, one receiving standard care (*n* = 407) and the other one enrolled in a PB-CM intervention (*n* = 396). They found no increase in gambling during and following the intervention (3 months post-intervention). Four years later, Petry and Alessi [89] reported similar findings in a smaller scale study with people using cocaine. Despite the publication of these findings, concerns remain over the risk of gambling as suggested by the number of studies that mention this risk and exclude potential participants on this basis. A recent systematic review [99] of individual differences in CM treatment response further confirms the need to address such concerns. In reviewing the evidence on PB-CM, it found that people with lower dopamine activity or people with reduced dopamine release capacity are most likely to be non-responders to PB-CM [99]. It also found that dopamine release capacity outperformed other predictors of PB-CM response such as demographic characteristics or substance use severity [99]. This contradicts the findings of Petry and her team [88, 89] and reinforces the need to engage in a more in-depth discussion of the ethical implications of PB-CM.

#### Deception by design

During our analysis, we identified two emerging ethical issues related to the potentially deceptive nature of PB-CM. The first issue concerns the central role played by the *appeal of winning* as opposed to the winnings themselves. This is unique to PB-CM because the literature on the ethics of incentives tends to focus on the winnings and their potential undue influence. For example, is it ethical to offer $50 to someone for immunizing their child? With PB-CM, the question is not whether the value amount is too much but rather if it is ethical to leverage the appeal of winning large value amounts to produce the greatest results at the lowest cost possible. In 2003, Petry and her team published a paper titled “Prize reinforcement contingency management for treating cocaine users: how low can we go, and with whom?” [76]. The title of this paper captures the goal of PB-CM: to reduce the costs of CM interventions and find a cheaper alternative to voucher-based CM that can yield similar (or even better) results. The second ethical issue concerns the technique known as “priming” [26]. As mentioned above, priming refers to a process whereby the researchers boost the appeal at the start of the intervention. In our sample, priming was done by providing a guaranteed incentive to offset the lower frequency and value amount of draws at the start or by adjusting the magnitude by increasing the probability of drawing a winning slip at the beginning of the intervention. Taken together, these two ethical issues have important implications for providers tasked with implementing and managing PB-CM who can find themselves in an ethical conundrum. On one hand, they want to optimize care for people who use substances and achieve greater health outcomes. On the other hand, using an intervention that relies on people *thinking* they will “win big” if they meet these outcomes when in fact the probabilities of winning are much lower than they appear can give rise to some important ethical and professional tensions.

#### Emphasizing individual behaviors

By design, PB-CM focuses primarily on individual behaviors as the source of the problem and the site of intervention. Aside from the information provided in each article to describe study participants, which we will discuss in the next section, PB-CM does not take into consideration broader social and structural determinants of health nor does it address the complexity of issues faced by people who use substances. In our sample, we noted only a few mentions of unemployment, mental illness, housing insecurity or homelessness, and poverty as having an impact on treatment adherence, abstinence, and engagement in care and counseling. PB-CM interventions are not designed to take into account the larger context in which people use substances. If and when they do, they tend to place the responsibility back onto the individual. For example, some studies included employment targets such as writing a resume [31, 74, 100], thus clearly placing the responsibility and solution at the level of the individual. Reasons for people who use substances to be unemployed are manifold and it is clear from the extensive body of research on the topic that not having a resume is far from being a major driver of unemployment [101]. Placing the emphasis on the individual to the detriment of social and structural determinants of health explains, in large part, why the effects of PB-CM are short-lived. Once the intervention stops, the behavior stops as well. This is consistent with the findings of a systematic review conducted by Benishek and colleagues on the use of PB-CM to reward abstinence [41]. They found that PB-CM increased abstinence in the short term but stopped producing effects after the intervention ended. At 6 months, they could no longer detect any effect from the intervention. The individual (and limited) focus of PB-CM combined with its short-term effects challenges the idea that deception is ethically justifiable because PB-CM is beneficial to people who use substances and highlights the need to provide patient-centered care, not behavioral-driven care.

#### Failing to address vulnerability and power dynamics

In our sample, we found that PB-CM researchers clumped people who use substances together and in the process did not account for and engage with factors such as socioeconomic status, race, and concurrent issues such as homelessness and mental health. This is a major gap because people living in poverty and experiencing housing insecurity (including homelessness), people living with a mental illness, and Black, Indigenous and People of Color (BIPOC) are overrepresented in PB-CM research. The sample of original studies included in this review totalled close to 6,500 participants. Of those participants, annual income averaged less than $20,000. Unemployment rates ranged from 12 to 76% across the studies. African-Americans were overrepresented and made up 30–77% of the research participants. There was also a strong representation of people experiencing homelessness and living with a mental illness with a number of studies focusing explicitly on PB-CM among these groups. Finally, it is important to note that 17% to 94% of research participants had been referred to the PB-CM intervention through their interactions with the legal system. Despite this, PB-CM interventions did not include safeguards to protect people who use substances who find themselves in a vulnerable position mentally, economically, socially, and/or structurally. As Voigt [102] points out, incentives such as PB-CM are notorious for targeting people who experience the greatest health and social inequities because they are not in a position to decline and often present complex issues that are “very costly and difficult-to-manage” [81]. In addition to the ethical concerns that arise from this phenomenon, two other concerns are unique to PB-CM and worth mentioning here. The first is the power dynamics that comes from having healthcare providers administer PB-CM as part of care. The second is the standard magnitude used in PB-CM. As pointed out by Adams and colleagues, magnitude should always be assessed in relation to socioeconomic status [59]. Not only does research on PB-CM fail to do this, but it also seeks to boost the appeal of winning while keeping the reward as low as possible. This additional element of deception combined with the individual focus of PB-CM, its short-term effects, and failures to address vulnerability and power in design and delivery further deepens the ethical concerns we have identified in our findings.

## Discussion

In this scoping review, we conducted a two-pronged analysis of the articles retrieved through a comprehensive search across key scholarly databases.

First, analyzed the characteristics of PB-CM interventions which were predominantly quantitative studies, mainly randomized controlled trials, aimed at studying the efficacy of PB-CM interventions. All of the interventions used a prize-draw format with a classic magnitude of 50%. Most of the interventions combined both negative and positive direction to target processes, behaviors, and/or outcomes among adults who use substances. They were designed according to a “certain chance” model, incentivizing a target immediately when it is reached using the same probability of winning. Finally, they used a variable schedule which was combined with an escalating and re-set technique. We found that studies were typically based on behavioral economics and failed to account for the shift in the science of addiction and most importantly, root causes that are now widely recognized such as trauma [103], social dislocation [104], desire [105], and the intersections of social, physical, economic, and policy environments that create contexts of substance use as well as related risks and harms [106]. Rather, they continued to see substance use, including opioid, alcohol, nicotine, stimulant, and benzodiazepine dependency as a matter of individual choice. It is notable that the majority of this research is conducted in the USA, a country that has “rejected harm reduction both rhetorically and substantively” for the past 40 years [107]. This is in stark contrast to other countries that have historically included harm reduction as part of their policy and practice approach to substance use. In Australia and Canada, for example, providers have been critical of PB-CM and have refused to use it in large part because of its abstinence focus and incompatibility with harm reduction [22, 44, 45].

Second, we attempted to identify the ethical implications discussed in these studies but found very little direct engagement with the range of ethical issues arising from PB-CM. In the absence of explicit ethical deliberation and guidance, we conducted an analysis to surface implicit ethical issues related to the deceptive nature of PB-CM, the predominant focus on individual behaviors (or ‘failings’) at the expense of social and structural determinants influencing these behaviors, the vulnerability (psychological, medical, economic, and social) of study participants, and failures to address power dynamics between providers who use PB-CM and their patients. Ethics in this literature is currently framed through research ethics and what is permissible when conducting studies about PB-CM. We do not question whether people who use drugs and other substances are able to consent to research, but study designs which intentionally target and manipulate the same neural pathways which regulate other ‘addictive’ behaviors (as evidenced by the exclusion of people diagnosed with a gambling addiction) in the context of healthcare delivery raises, or should raise, considerably more discussion in the research ethics literature. Furthermore, when the ultimate goal is to translate this research into interventions and programming where healthcare providers are tasked with administering the PB-CM, a clinical ethics lens is needed. Through a clinical ethics lens, it is clear that PB-CM raises questions related to potential harms, undue influence, equity, relational care, and best practices when working with people who use substances. If we add a lens of public health ethics, we can identify additional questions related to resource allocation, social determinants of health, and the role of governments in creating health disparities and conditions favorable to the emergence of public crises such as the ongoing overdose crisis.

The findings of the scoping review are consistent with recent research, which suggests that providers who are tasked with implementing PB-CM worry about the impact of repetitive disappointment from either winning low-value slips (or non-winning slips) or being denied the opportunity to draw [22]. As such, they find themselves in a position where they have to work to “support clients through their own disappointment” and to minimize the potential harms of clients feeling “betrayed” [22]. Their experiences point to important gaps between PB-CM research and PB-CM *in practice*. In practice, for example, providers face added responsibilities such triaging clients who are eligible for the intervention and those who are not, communicating and managing expectations around the draw, maintaining boundaries and strict rules (e.g., draw upon success), and addressing issues around autonomy, power imbalance, and fairness [22]. However, as shown in this paper, PB-CM research does not consider the contextual or interpersonal dimensions of the intervention itself, nor does it reflect an understanding of potential implications for both providers and patients. In our research, for example, providers wanted to “value effort over success, support over reward, honesty over deceit, and certainty over probability and variability” [22]. This was especially true when working with patients who experienced multiple vulnerabilities and complex intersecting health needs. It comes as no surprise that PB-CM remains one of “the least implemented” of all empirically based interventions in substance use [20]. Here, the lack of uptake should be understood as an indication that more engagement is needed, not interpreted as a sign of resistance on the part of providers who question PB-CM. Generally speaking, they do not question the evidence behind PB-CM; they question the ethics of it.

In terms of future directions for PB-CM research, policy, and practice, we call for greater ethical analysis beyond whether to exclude research participants with a gambling issue and greater attention to ethical implications of interventions designed to advance the science while yielding incremental changes in the health and well-being of people who use substances. Additionally, we may wish to question the ethics of continuing to develop short-term interventions to complex issues that are rooted in social and structural inequities. At the very least, such research seeking to develop interventions would benefit from greater consultation with people who use substances who have advocated for inclusion and representation in research about them [108–110]. There is a considerable body of research about the meaningful engagement of people who substances [111, 112]. We further call for more implementation research to explore the practice implications of using PB-CM. Finally, we call for greater ethical analysis to assess the merits of PB-CM as a policy intervention on a system-level scale. We are acutely aware of the impact of the overdose crisis and of the COVID-19 pandemic on the health and wellness of people who use substances, especially physical distancing requirements and restrictions to addictions treatment and harm reduction services which have increased overdoses in many jurisdictions. We call for greater scrutiny over the use of limited resources to support PB-CM interventions, focused on short-term individual behavior change, instead of structural interventions which will have broader impacts on health outcomes and social determinants of health.

### Strengths and limitations

This study has several strengths, including that it is the first comprehensive scoping review of design and ethical issues in the PB-CM literature. A rigorous scoping review methodology was used, including accessing key databases with the aid of a specialized librarian, and we synthesized the literature with an attention to the needs of people who use substances, healthcare providers, researchers, and policy-makers. We identified a near absence of formal ethical engagement and debate about a practice of PB-CM with implications for research ethics, clinical ethics, and public health ethics. The limitations of the study are related to the nature of scoping reviews. While over half of the studies we reviewed were randomized controlled trials (*n* = 30 or 55.7%), we did not conduct quality appraisals of the interventions or attempt to pool across studies because this would not have been relevant to the objectives of our review. These randomized controlled trials reported on the outcomes of PB-CM interventions with very limited discussion of ethics beyond reporting whether research ethics clearance was secured for the trials and exclusion criteria, and did not test outcomes related to ethics (e.g., the use of different ethical decision-making aids in retaining patients). Moreover, they did not provide insights into routine care and programming in real-world care scenarios which are imbued with greater ethical complexity. Although this may be a limitation associated with the RCT design itself, it creates barriers to evidence-based clinical practice. If the goal is to implement PB-CM in practice, implementation science may be best suited for future research and will help address the issues discussed in this paper. As suggested in this scoping review, it is important to consider how PB-CM *translates into practice* in order to understand if this intervention “works” in the context of substance use care and treatment.

## Conclusion

This scoping review offers important insights into the research on PB-CM and raises ethical questions that are not only valuable to healthcare providers who are working with people use substances in clinical practice, but also those working to tackle the dual crisis of opiate and stimulant use—a crisis fueled by widening health and social inequities. As such, they are also valuable for other health and allied health providers potentially tasked with administering PB-CM in community and public health settings. With the rising uptake of incentives in health care, we have observed a trend towards monetizing healthcare delivery even in jurisdictions with universal insurance coverage. The promise of easy fixes offered by incentives such as PB-CM is appealing to a range of health systems stakeholders regardless of the short-term nature of the potential “benefits.” The question here is not whether PB-CM works, but rather if they are appropriate and ethical when caring for people who use substances. Having identified a critical gap in the literature, it is our hope that our findings can inform future research and dialogues to further tease out the ethical issues associated with PB-CM.

## Data Availability

The datasets used and/or analyzed during the current study are available from the corresponding author on reasonable request.

## Abbreviations

CM: Contingency management
PC-CM: Prize-based contingency management
